# A Device for Isolation of Selected Single Adherent Cells

**DOI:** 10.1155/2022/4303586

**Published:** 2022-12-15

**Authors:** Wang-Ying Dai, Jiang-Bo Guo, Xi Chen, Zong-Ping Luo

**Affiliations:** Orthopaedic Institute, Department of Orthopaedics, The First Affiliated Hospital of Soochow University, 708 Renmin Rd, Suzhou, Jiangsu 215007, China

## Abstract

In recent years, extensive research has been focused on the field of single cell analysis. The isolation of single cells is the first step in this type of research. However, the techniques used for direct isolation and acquisition of single adherent cells are limited. Here, we present a method of obtaining selected single adherent cells using a separation device. Compared with other single cell isolation methods, this method has the advantages of simple operation, low cost, minimal cell damage, and preservation of cell morphology. Our methodology is, therefore, suitable for the collection of selected single adherent cells.

## 1. Introduction

Recently, extensive research in the fields of embryonic development [1], tumor biology [2], drug research [3], and stem cell biology [4], has been focused on single cell analysis in order to comprehensively understand the differences among single cells and their biological characteristics.

At present, researchers isolate single cells mainly using microfluidic chip technology and fluorescence activated cell sorting (FACS) technology [5, 6]. These techniques isolate single cells from cell suspensions [7, 8]. The laser capture microdissection (LCM) technique is also used for isolating single cells from tissues [9–11]. These techniques, however, cannot be used directly to isolate single cells from adherent cell populations.

This study proposes a single cell isolation technique requiring a microtube and a stereotaxic apparatus to enable convenient and damage free selection and isolation of single cells from adherent cell populations. If the technology can be successfully developed, it will be beneficial to the further study of adherent single cells.

## 2. Materials and Methods

### 2.1. Polyacrylamide Hydrogel Synthesis

The hydrogel was synthesized using a western blot gel holder cassette (Bio-Rad, CA, USA). Two glass plates having a gap of 1.0 mm that form the gel holder cassette were washed and dried. 40% acrylamide (Sigma, USA), 2% methylidene bisacrylamide (Sigma, USA), and proportionate volume of ultrapure water were mixed together. 10% ammonium persulfate (APS Sigma, USA) and tetramethylethylenediamine (TEMED, Thermo Fisher Scientific, USA) were added to the above mixture and mixed rapidly to initiate the reaction. The mixture was added between the two glass plates and allowed to stand at room temperature for 2 hours to form a gel. Four different types (A, B, C, and D) of polyacrylamide hydrogels were synthesized using varying compositions of the reagents (Table 1).

### 2.2. Polyacrylamide Hydrogel Processing for Cell Seeding

After the hydrogel was formed, it was washed twice with phosphate buffer saline (PBS) to remove any unreacted reagents. The gel was soaked in amicrobic PBS for 7 days, followed by irradiation with an ultraviolet lamp for 20 min. The PBS was removed, and the polyacrylamide hydrogel was treated with a 0.75 mg/mL sulfo-SANPAH crosslinker solution (Sigma, USA) for 1 hour. Ultraviolet irradiation was used for 20 minutes again. After the end of the irradiation, the gel was allowed to stand for 1 hour. The sulfo-SANPAH solution was removed and the hydrogel was incubated with serum-free medium for at least 6 hours. Finally, the cells were seeded on the hydrogel for culture.

### 2.3. Cell Culture

Bone marrow stem cells (BMSCs) cells with green fluorescent protein (GFP) were purchased from Cell Storage Center of Otwo (Guangzhou, China). Cells were cultured in *α*-Minimum Essential Medium (*α*-MEM, Gibco, Grand Island, NY) with 10% fetal bovine serum (Gibco, Grand Island, NY) and 1% streptomycin/penicillin (Gibco, Grand Island, NY) and maintained at 37°C under 5% CO_2_. The medium was changed every 3 days during the incubation period. Before the experiment, the cells were then transferred into 6-well plates at a cell density of 1 × 10^4^. After 24 hours of culture, the cells were collected.

### 2.4. Cell Collection

**Figure 1:**
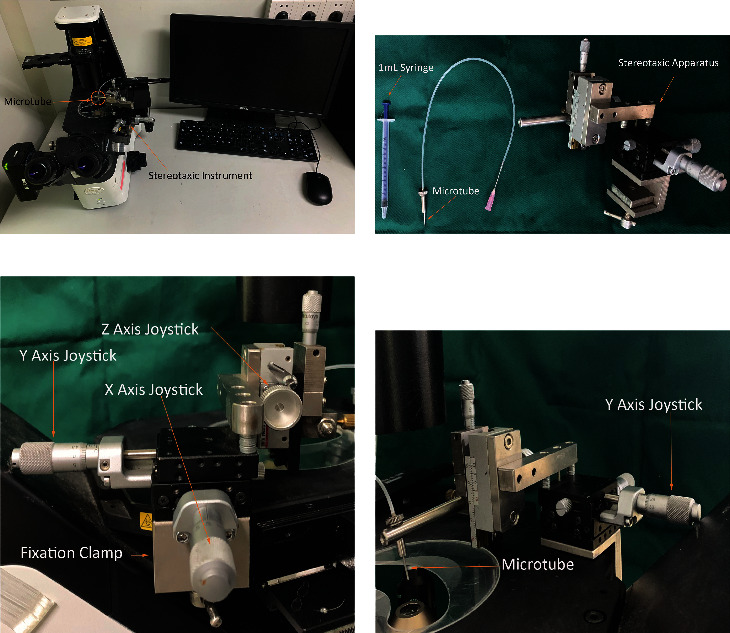
Separation device for single adherent cells. (a) The device needs to be used with a computer monitor and an inverted microscope, (b) the device includes 1 mL syringe, microtube, and stereotaxic apparatus, (c) and (d) the stereotaxic apparatus is fixed onto the inverted microscope.

**Figure 2:**
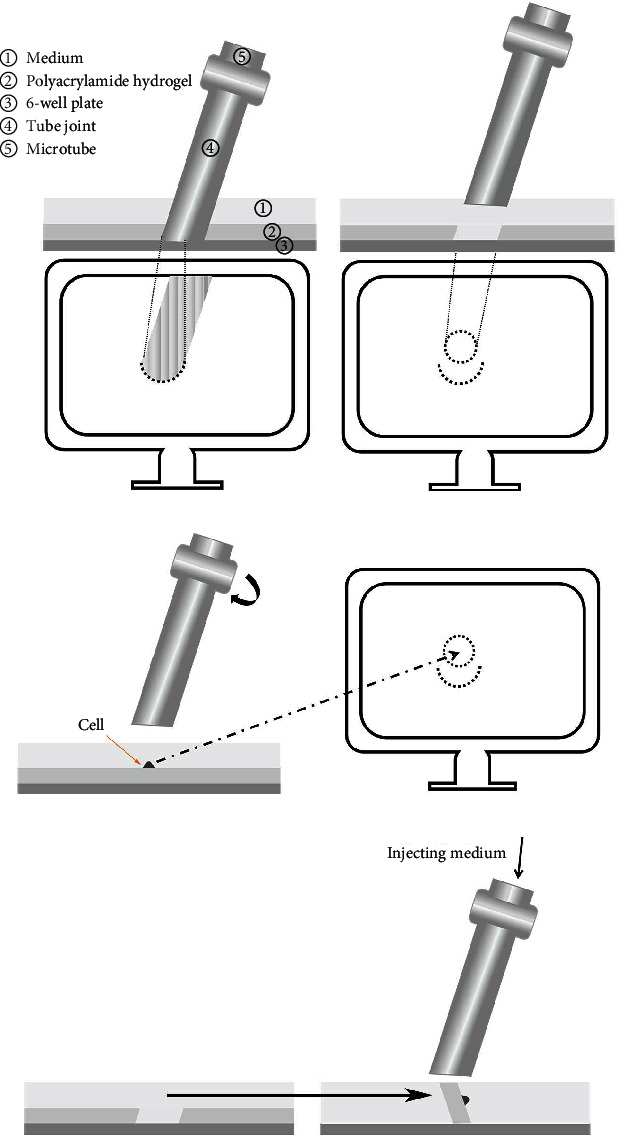
Schematic diagram of single cell isolation. (a) The microtube was adjusted to the appropriate tilt angle, and the display position of the microtube was marked on the computer screen. (b) The specific area to be targeted was identified prior to cell isolation and marked on the screen. (c) The target cell was moved to the marked area, and after the microtube was placed at the designated position, the target cell along with the hydrogel surface on which it is adherent was separated by rotating the microtube. (d) The cell in the microtube was gently pushed out using the medium (Pointed at by the arrow).

A microtube (diameter is 500 *μ*m) and an inverted microscope were used to isolate the target cells. To begin with, the stereotaxic apparatus was fixed (Figure 1). The microtube was then adjusted to the appropriate tilt angle (5° to 10°), and the position of the microtube was marked on the computer screen. The specific area to be targeted was then identified prior to cell isolation. Next, the target cell was moved to the marked area, and after the microtube was placed at the designated position, the target cell along with the hydrogel surface on which it is adherent was separated by rotating the microtube (Figure 2). Finally, the cell in the microtube was gently pushed out using the *α*-MEM, and the isolated cell was placed in a new 6-well plate.

### 2.5. Isolation Success Rate of Polyacrylamide Hydrogel

In order to test the isolation efficiency of polyacrylamide hydrogel, we trained 3 technicians to independently test the cells isolated on each of the four types of polyacrylamide hydrogel (Table 1). Three measurements were taken to obtain an average value for each polyacrylamide hydrogel. Three technicians who were blinded to the experimental conditions independently performed the tests to obtain the results. Each technician took 10 gel on each sample to calculate the success rate.

### 2.6. Statistical Analysis

Experimental data were analyzed using the SPSS 20.0 software and expressed as the mean ± SEM. The statistical significance was determined with one-way analysis of variance (ANOVA) test, followed by Dunnett's LSD post-hoc testing to calculate the longitudinal differences between the groups. A value of *P* < 0.05 was considered statistically significant.

## 3. Results

### 3.1. Cell Collection

We first tested the isolation rate from the four types of polyacrylamide hydrogels used was first assessed. The results showed that the single cell isolation rate from the group D hydrogel was the highest (*P* < 0.01). Therefore, the reagent composition used in the group D hydrogel is considered the best choice for this experiment.

**Figure 3:**
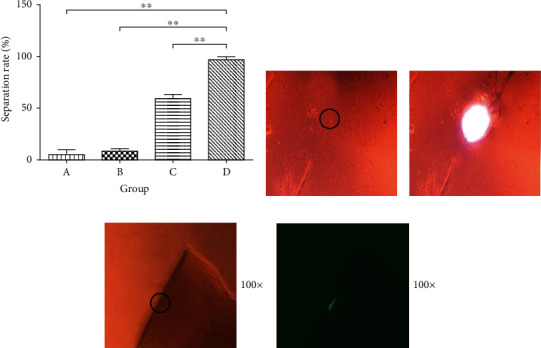
Isolation of adherent single cells. (a) Single cell isolation efficiency of the four types polyacrylamide hydrogels. (b) and (c) The polyacrylamide hydrogels before and after single cell separation, respectively. (d) The single cell was examined under a fluorescence microscope. (e) The isolated fluorescent single cell, ^∗∗^*P* < 0.01 compared with for D group.

The experimental results of the cell collection setup (Figures 1 and 2) show that by comparing the position of the polyacrylamide hydrogel before and after the collection, it can be tentatively confirmed that the target cells and hydrogels of the corresponding regions have been obtained. Also, the single cells attached to the surface of the hydrogel can be seen under the inverted microscope, and the cells with green fluorescence can be seen under a fluorescence microscope (Figure 3).

### 3.2. Cell Culture

**Figure 4:**
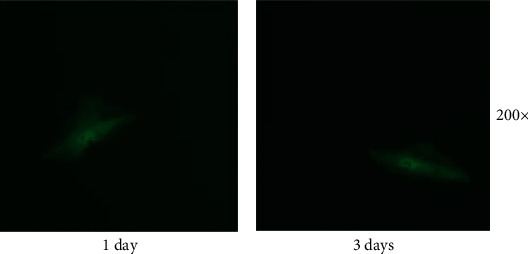
Fluorescent images of cell culture. Images of cells examined under a fluorescence microscope at 1(a) and 3(b) days after isolation.

In order to determine whether the isolated single cells can be cultured, they were cultured in a 6-well plate and images were captured on the 1st and the 3rd day. The results showed that the morphology of the isolated single cells was still fusiform. Although no division or proliferation was observed, cells moved freely. These results thereby prove that the isolated cells remained viable for culture (Figure 4).

## 4. Discussion

### 4.1. Polyacrylamide Hydrogel

In this methodology, we used polyacrylamide hydrogel as the substrate for cell culture. The polyacrylamide hydrogel prepared in the experiment is a transparent and nonfluorescent hydrogel [12]. Under the microscope, the hydrogel allows light to transmit, thereby enabling better visualization of target cells. Past studies have found polyacrylamide hydrogels to have good biocompatibility [13, 14]. In this experiment, we used sulfo-SANPAH crosslinker to treat the surface of the hydrogel to increase cell adhesion [15]. So, all of the above conditions make cells grow better on hydrogels.

### 4.2. Isolation of Selected Single Cells

In the experiment, polyacrylamide hydrogels with different compositions have different cell isolation efficiencies. Our results showed that the isolation efficiency of group D was the highest, but it was not 100% successful. The possible reasons for the incomplete success are as follows: (1). different types of hydrogels having different modulus of elasticity, resulting in different degrees of isolation difficulty; (2) insufficient technical proficiency of technicians.

The results of this experiment show that the isolated single cells can continue to grow in culture. The cells obtained by this technology can survive, which will create conditions for subsequent analysis and application.

### 4.3. Comparative Analysis of the Proposed Technique with Existing Techniques

At present, the isolation technique of single cells is mainly divided into two categories: one is the isolation techniques of nonadherent single cells and another is the isolation techniques of adherent single cells. The former include microfluidic chip technology, FACS, and LCM [7, 16]. Micromanipulation, photopolymerization technique, and ferromagnetic micropallets array belong to the latter [7]. In recent years, many devices have appeared on the market (ALS CellCelector, Yamaha Cell Handler, CellSorter), but they are all very expensive.

FACS sorts cells in a particular population by means of cell surface markers or cell characteristics. FACS can obtain single cells in large quantities. This technique is feasible, and the experimental standards are easy to unify. It is a widely used method, but cells must be in suspension and this affects the cell state to some extent [7]. On the other hand, the technique applied in this study is suitable for the adherent heterogeneous single cells with unknown markers.

LCM is capable of isolating single cells directly from tissue. However, the high precision required for cutting the thin tissue slices proves to be a limitation of this technique. The quality of sequencing data obtained from microdissected single cells is relatively poor, and the tissue needs to be sliced and fixed [8]. In recent years, it has also been used for cell culture [17]. The proposed technique neither requires the target cells to be isolated by microdissecting tissues nor requires the cells to be fixed. Therefore, it overcomes the limitations of the former technique and can be used for sequencing and transcriptome analysis.

*Microfluidic chip technology*. The core of the microfluidic device is a channel that allows only a single cell to pass through, and its diameter can be adjusted according to the size of the cell. Also, the channel can be modified according to the cell type that needs to be sorted and detected. The incorporation of the microfluidic device into the biochip reduces the amount of fluid, thus increasing the concentration of the sample [5, 18]. However, due to its high cost, this technique cannot be used extensively for practical applications. The cost issue has also been adequately addressed by our proposed technique in that while the technique is highly feasible, the equipment cost is low.

At present, the ferromagnetic micropallets array is used to isolate adherent single cells [19]. This technique is convenient and accurate in the selection and isolation of single cells. However, the method has limitations in neural cell culture. For example, nerve cells may not be able to fully extend in the micropallet. At the same time, there is lack of contact among cells.

Photopolymerization technique has also been proposed as a single cell isolation technique by some researchers. In this technique, single cells can be separated by photopolymerized hydrogels [20]. The separation method, however, may have additional effects on cells during hydrogel polymerization.

While there are micromanipulation methods [21–23], such as using a pipette to aspirate cells, this methods can easily damage cells. On the other hand, our technique can ensure the integrity of the cell, thereby making it the technique of choice for selecting a single adherent cell with special phenotype.

Our technique has the same limitation as the isolation techniques of adherent single cells: the isolation is not as effective when the cells overlap. Further study is required to develop a technique capable of overcoming these limitations.

## 5. Conclusions

In this study, a device for isolation of selected single adherent cells was proposed. The method has the advantages of maintaining cell morphology, minimal cell damage, ease of operation, and low cost. This technique which can directly separate a single target cell from numerous adherent cells may become a useful tool in the field of single cell analysis.

## Figures and Tables

**Table 1:** Different compositions of Polyacrylamide hydrogel.

Group	40% acrylamide (mL)	2% Methylidene Bisacrylamide (mL)	Ultrapure water (mL)	10% APS (*μ*L)	TEMED (*μ*L)	Elastic modulus (kPa)
A	0.75	0.45	4.8	60	6	4.47 ± 3.98
B	1.5	0.3	4.2	60	6	10.61 ± 4.01
C	1.5	0.9	3.6	60	6	34.88 ± 4.33
D	1.5	3.3	1.2	60	6	60.42 ± 5.40

## Data Availability

The data used to support the findings of this study are included within the article.

## References

[1] Alemany A., Florescu M., Baron C. S., Peterson-Maduro J., van Oudenaarden A. (2018). Whole-organism clone tracing using single-cell sequencing. *Nature*.

[2] Zhang Q., He Y., Luo N. (2019). Landscape and dynamics of single immune cells in hepatocellular carcinoma. *Cell*.

[3] Wu H., Wang C., Wu S. (2017). Single-cell sequencing for drug discovery and drug development. *Current Topics in Medicinal Chemistry*.

[4] Wells C. A., Choi J. (2019). Transcriptional profiling of stem cells: moving from descriptive to predictive paradigms. *Stem Cell Reports*.

[5] Junkin M., Tay S. (2014). Microfluidic single-cell analysis for systems immunology. *Lab on a Chip*.

[6] Milliron H. Y., Weiland M. J., Kort E. J., Jovinge S. (2019). Isolation of cardiomyocytes undergoing mitosis with complete cytokinesis. *Circulation Research*.

[7] Saliba A. E., Westermann A. J., Gorski S. A., Vogel J. (2014). Single-cell RNA-seq: advances and future challenges. *Nucleic Acids Research*.

[8] Shapiro E., Biezuner T., Linnarsson S. (2013). Single-cell sequencing-based technologies will revolutionize whole-organism science. *Nature Reviews. Genetics*.

[9] Todd R., Lingen M. W., Kuo W. P. (2002). Gene expression profiling using laser capture microdissection. *Expert Review of Molecular Diagnostics*.

[10] Schutze K., Posl H., Lahr G. (1998). Laser micromanipulation systems as universal tools in cellular and molecular biology and in medicine. *Cellular and Molecular Biology (Noisy-le-Grand, France)*.

[11] Burgemeister R. (2005). New aspects of laser microdissection in research and routine. *The Journal of Histochemistry and Cytochemistry*.

[12] Kandow C. E., Georges P. C., Janmey P. A., Beningo K. A. (2007). Polyacrylamide hydrogels for cell mechanics: steps toward optimization and alternative uses. *Methods in Cell Biology*.

[13] Tuson H. H., Renner L. D., Weibel D. B. (2012). Polyacrylamide hydrogels as substrates for studying bacteria. *Chem Commun (Camb)*.

[14] Risbud M. V., Bhonde R. R. (2000). Polyacrylamide-chitosan hydrogels: in vitro biocompatibility and sustained antibiotic release studies. *Drug Delivery*.

[15] Pelham R. J., Wang Y. (1997). Cell locomotion and focal adhesions are regulated by substrate flexibility. *Proceedings of the National Academy of Sciences of the United States of America*.

[16] Tang F., Lao K., Surani M. A. (2011). Development and applications of single-cell transcriptome analysis. *Nature Methods*.

[17] Brasko C., Smith K., Molnar C. (2018). Intelligent image-based in situ single-cell isolation. *Nature Communications*.

[18] Schoeman R. M., Kemna E. W., Wolbers F., van den Berg A. (2014). High-throughput deterministic single-cell encapsulation and droplet pairing, fusion, and shrinkage in a single microfluidic device. *Electrophoresis*.

[19] Salazar G. T., Wang Y., Young G. (2007). Micropallet arrays for the separation of single, adherent cells. *Analytical Chemistry*.

[20] Negishi R., Iwata R., Tanaka T. (2019). Gel-based cell manipulation method for isolation and genotyping of single-adherent cells. *Analyst*.

[21] Morris J., Singh J. M., Eberwine J. H. (2011). Transcriptome analysis of single cells. *Journal of Visualized Experiments*.

[22] Citri A., Pang Z. P., Sudhof T. C., Wernig M., Malenka R. C. (2012). Comprehensive qPCR profiling of gene expression in single neuronal cells. *Nature Protocols*.

[23] Zeng J., Mohammadreza A., Gao W. (2014). A minimally invasive method for retrieving single adherent cells of different types from cultures. *Scientific Reports*.

